# Novel gene signatures for prognosis prediction in ovarian cancer

**DOI:** 10.1111/jcmm.15601

**Published:** 2020-07-14

**Authors:** Mingyang Bao, Lihua Zhang, Yueqing Hu

**Affiliations:** ^1^ State Key Laboratory of Genetic Engineering Institute of Biostatistics School of Life Sciences Fudan University Shanghai China; ^2^ Department of Gynecology Jiangsu Cancer Hospital Jiangsu Institute of Cancer Research Nanjing Medical University Affiliated Cancer Hospital Nanjing China; ^3^ Shanghai Center for Mathematical Sciences Fudan University Shanghai China

**Keywords:** LASSO Cox, multi‐gene signature, ovarian cancer, single‐gene signature

## Abstract

Ovarian cancer (OV) is one of the leading causes of cancer deaths in women worldwide. Late diagnosis and heterogeneous treatment result to poor survival outcomes for patients with OV. Therefore, we aimed to develop novel biomarkers for prognosis prediction from the potential molecular mechanism of tumorigenesis. Eight eligible data sets related to OV in GEO database were integrated to identify differential expression genes (DEGs) between tumour tissues and normal. Enrichment analyses discovered DEGs were most significantly enriched in G2/M checkpoint signalling pathway. Subsequently, we constructed a multi‐gene signature based on the LASSO Cox regression model in the TCGA database and time‐dependent ROC curves showed good predictive accuracy for 1‐, 3‐ and 5‐year overall survival. Utility in various types of OV was validated through subgroup survival analysis. Risk scores formulated by the multi‐gene signature stratified patients into high‐risk and low‐risk, and the former inclined worse overall survival than the latter. By incorporating this signature with age and pathological tumour stage, a visual predictive nomogram was established, which was useful for clinicians to predict survival outcome of patients. Furthermore, SNRPD1 and EFNA5 were selected from the multi‐gene signature as simplified prognostic indicators. Higher EFNA5 expression or lower SNRPD1 indicated poorer outcome. The correlation between signature gene expression and clinical characteristics was observed through WGCNA. Drug‐gene interaction was used to identify 16 potentially targeted drugs for OV treatment. In conclusion, we established novel gene signatures as independent prognostic factors to stratify the risk of OV patients and facilitate the implementation of personalized therapies.

## 1 INTRODUCTION

Ovary cancer (OV) is one of the most common gynaecological tumours with the fourth highest morbidity and the third highest mortality worldwide.^1^ In China, the mortality rate of OV ranks second in gynaecological tumours and shows an upward trend while the incidence rate keeps declining.^2^ Due to poor prognosis, the proportion of female deaths caused by OV is greater than that of female cancers caused by OV in all malignancies in the United States.^3^ The main reason for these observations is that more than 70% cases with OV are not diagnosed until the tumour has progressed to advanced stages (stage III–IV; International Federation of Gynecology and Obstetrics, FIGO).^4^

At present, effective screening test for early OV detection has not been accessible. Biological markers such as the carbohydrate antigen 125 (CA125) and human epididymis protein (HE4) are widely used in clinical diagnosis.^5^, ^6^, ^7^ However, the serum CA125 level is not specific for OV because its elevation may result from menstruation, benign gynaecological diseases and other cancers in spite of high sensitivity.^8^ On the other hand, HE4 has reliable specificity but poor sensitivity.^5^, ^9^ What's more, the prognosis cannot be predicted although the combination of these biomarker levels improves diagnostic accuracy. Therefore, it is necessary to explore gene signatures associated with prognostic prediction from the potential mechanism of OV progression.

The G2/M DNA damage checkpoint serves to prevent the cell with DNA damage from entering mitosis (M‐phase) during cell cycle.^10^ In most tumours, upstream G1/S checkpoint is inactivated due to the loss of function of tumour suppressor genes, which strengthens their survival ability. Meanwhile, it means that tumour cells mainly rely on the G2/M checkpoint to avoid factors that disrupt genome stability. Furthermore, previous researches have shown robust correlations between G2/M cell cycle arrest and prognosis for multiple cancers, including OV.^11^, ^12^, ^13^

Nevertheless, survival varies by category of OV. Epithelial cancers are the most common OV types.^3^ Serous carcinoma, the most common epithelial subtype by histological classification, is mainly diagnosed at late stage and possesses aggressive nature of high grade.^14^ Both advanced stage and high grade are important factors associated with worse prognosis.^15^, ^16^ Prognostic predictors need to be further developed, especially for patients with these poor outcome indicators. Previous studies have identified several potential genes for predicting the prognosis of OV but their comprehensiveness and clinical application remain limited.^17^, ^18^, ^19^ In this study, we discovered that differential expression genes (DEGs) between tumour and normal tissues were most significantly enriched in G2/M checkpoint signalling pathway based on the several data sets in the Gene Expression Omnibus (GEO) and The Cancer Genome Atlas (TCGA) data sets. The multi‐gene and single‐gene signatures were constructed on genes related to G2/M checkpoint and validated in cohorts of OV patients.

## 2 MATERIALS AND METHODS

### 2.1 Data collection

We searched for data sets related to OV from the GEO database (http://www.ncbi.nlm.nih.gov/geo/) with the Mesh terms ‘ovary neoplasms’ and ‘human’. A further filter was performed with organism ‘Homo sapiens’, study type ‘Expression profiling by array’ and samples count ‘Higher than ten’. According to the systematic screening strategy, a total of eleven data sets were eventually included in this study. Eight data sets were used to screen DEGs, including GSE105437, GSE54388, GSE69428, GSE14407, GSE12470, GSE4122, GSE10971 and GSE26712. GSE23554, GSE14764 and GSE63885 were applied at the validation stage. Twenty‐six samples from GSE63885 with incomplete survival data were removed. Detailed information was shown in Table 1. Raw data were processed with robust multi‐array average expression measure (RMA) background correction, log2 transformation and normalization. Moreover, expression profiling and clinical information of the samples with complete prognostic data were downloaded from the TCGA‐OV data set (https://cancergenome.nih.gov/). The gene list of hallmark gene sets ‘HALLMARK G2M CHECKPOINT’ was downloaded from the Gene Set Enrichment Analysis (GSEA) database (http://software.broadinstitute.org).

**TABLE 1:** Characteristics of GEO data sets included in the study

Data set ID	Platform ID	Country	Number of samples	
			Tumour	Normal
GSE105437	GPL570	South Korea	10	5
GSE54388	GPL570	USA	16	6
GSE69428	GPL570	USA	10	10
GSE14407	GPL570	USA	12	12
GSE12470	GPL887	Japan	43	10
GSE4122	GPL201	USA	32	14
GSE10971	GPL570	Canada	13	24
GSE26712	GPL96	USA	185	10
GSE23554	GPL96	USA	28	‐
GSE14764	GPL96	Germany	80	‐
GSE63885	GPL570	Poland	101	‐

Abbreviations: GPL, Gene Expression Omnibus Platform; GSE, Gene Expression Omnibus Series.

### 2.2 DEGs identification

GEO2R (http://www.ncbi.nlm.nih.gov/geo/geo2r/) was used to detect DEGs in each GEO data set. *P* values and log fold change (FC) of duplicate genes were averaged based on the ‘limma’ package in R.^20^ Significant DEGs were defined as those with adjusted *P* < .05 and |log FC| ≥ 1 and were ranked by the logFC in each microarray data set. The results of eight series accessions were integrated through the ‘RobustRankAggreg (RRA)’ R package to select the most significant DEGs.^21^

### 2.3 Enrichment analyses

Gene Ontology (GO) enrichment and Kyoto Encyclopedia of Gene and Genomes (KEGG) pathway analyses were conducted using Metascape (http://www.metascape.org/), a powerful annotation tool for gene function analyses.^22^ We also performed enrichment analysis using the hallmark gene sets as the reference gene set. In addition, protein‐protein interaction (PPI) enrichment analysis was carried out. The Molecular Complex Detection (MCODE) algorithm was applied to identify densely network components.

### 2.4 Gene set enrichment analysis (GSEA)

We utilized javaGSEA 4.0.3 to perform GSEA within the above microarray data to analyse the difference between tumour and normal tissues. The most significant hallmark gene set in the enrichment analysis was selected as the reference gene set.

### 2.5 Construction of the prognostic gene signature

The genes in the hallmark gene set ‘HALLMARK G2M CHECKPOINT’ in which the DEGs were enriched most significantly were considered as candidate biomarkers. LASSO (least absolute shrinkage and selection operator) Cox regression model was used to construct multi‐gene signature for predicting OV prognosis from these candidate biomarkers.^23^ Based on the ‘glmnet’ R package, the model was applied to the expression matrix of candidate genes and the optimal value of the penalty parameter λ was selected to calculate the coefficient of each gene constituting prognostic signature. The sum of the product of these coefficients and gene expression for each sample, defined as the risk score of the prognostic gene signature, was used to evaluate the prognostic risks.

### 2.6 Prognostic value estimation of the multi‐gene signature

TCGA‐OV cohort was considered internal set, and GSE26712, GSE63885 and GSE14764 were deemed external sets for prognostic value estimation. The samples from each data set were divided into high‐risk and low‐risk groups according to the median risk score. Then, Kaplan‐Meier (KM) survival analysis was performed to estimate prognostic value of the gene signature. The prediction accuracy was assessed through time‐dependent receiver operating characteristic (ROC) curves and area under the curve (AUC) for 1‐year, 3‐year and 5‐year overall survival.^24^ Furthermore, subgroup analysis was conducted to determine independence of prognostic model from other clinicopathological features. The samples from GSE14764, GSE23554, GSE26712 and GSE63885 were integrated and stratified into various subgroups according to clinicopathological characteristics. In terms of residual tumour, patients were divided into residual tumour <1 cm and residual tumour ≥1 cm. Given the pathological grade, histology and chemotherapy, we selected the most common subtypes: high grade, serous carcinoma and response to chemotherapy. KM survival analysis was performed to examine prognostic significance in each subgroup. Comparisons between our G2/M checkpoint‐related multi‐gene signatures and other biomarker‐based models^19^, ^25^ were conducted with univariate Cox regression analysis and were assessed by the concordance index (C‐index) in the internal and external sets. The average C‐indices weighted by sample sizes were regarded as the representative ones of three external sets. The larger C‐index indicated the more accurate prognostic prediction.

### 2.7 Multivariate Cox regression analysis

Clinicopathological variables and risk score were included in multivariate Cox regression to determine which were significant prognostic factors. The result was shown in a forest plot using the ‘forestplot’ package in R. According to the regression coefficient, every independent variable corresponded to a point at each value. A total point was equal to the sum of the points of all independent variables for each patient. The relationship between the total points and the probability of the outcome event was visualized on the nomogram to predict 1‐year, 3‐year and 5‐year overall survival through the ‘rms’ and ‘regplot’ R package. The performance of the nomogram was measured by C‐index with 1000 bootstrap resampling for a relative correction. Calibration curves for 3‐year and 5‐year survival were subsequently drawn to investigate the closeness between nomogram‐predicted overall survival and the actual outcome. Diagonal considered as a reference represents the best prediction.

### 2.8 Prognostic values estimation of single‐gene signatures

From the multi‐gene signature, we selected the genes with prognostic significance and closely related to risk stratification in the TCGA cohort as simplified signatures for prognosis prediction. Differences in expression levels between OV and normal tissues were investigated based on the Gene Expression Profiling Interactive Analysis (GEPIA) (http://gepia.cancer‐pku.cn/index.html), an online database that included enormous samples across 33 different types of cancer.^26^ Analysis of gene expression in different subtypes of OV was further performed on the Oncomine database (https://www.oncomine.org/resource/login.html). Then, overall survival and disease‐free survival analysis were carried out to validate prognostic value of single‐gene signatures for prognosis prediction in the GEPIA database.

### 2.9 Correlation between signature genes and clinical characteristics

To further investigate the correlation between signature genes and clinical characteristics, we combined expression profiles of robust DEGs from RRA analysis and clinical data in TCGA to perform weighted gene co‐expression network analysis (WGCNA).^27^ ‘WGCNA’ R package was used to establish the topological overlap matrix based on the adjacent matrix composed of gene expression and clinical traits. Genes were classified into several modules associated with clinical traits according to dissimilarity measure. The correlations between genes in each module and clinical characteristics were identified by gene significance (GS) and module membership (MM).

### 2.10 Drug‐signature gene interaction

We searched for potential drugs response to promising targets G2/M checkpoint signalling pathway to which all genes in the multi‐gene signature were related. Drug‐Gene Interaction Database (DGIdb; http://www.dgidb.org) was used to explore interactions between drugs and eight signature genes. The interaction network was constructed by the online tool STITCH (http://stitch.embl.de).

### 2.11 Statistical analysis

Statistical analyses were conducted by online resources and R software 3.6.1. In brief, limma procedure was used to investigate differences in gene expression in GEO2R and the accumulative hypergeometric distribution was applied to carry out pathway and process enrichment analysis in Metascape. Terms with a *P*‐value <.01, a minimum count of 3 and an enrichment factor >1.5 (the enrichment factor was the ratio between the observed counts and the counts expected by chance) were collected and grouped into clusters based on their membership similarities. The *P* values obtained in the above two steps were adjusted by the Benjamini‐Hochberg procedure. Student's *t* test or one‐way ANOVA was performed to compare mRNA expression if the data were normally distributed; otherwise, Wilcoxon or Kruskal‐Wallis test was conducted for comparisons. The two‐sided log‐rank tests were performed to analyse survival differences between the high‐risk and low‐risk groups when KM survival curves were drawn based on the ‘survival’ and ‘survminer’ package in R. Univariate and multivariate Cox proportional hazard models were built to estimate the hazard ratios of prognostic factors. *P* < .05 was considered as statistical significance (*, *P* < .05).

## 3 RESULTS

### 3.1 Identification of integrated DEGs by the RRA method

**FIGURE 1:**
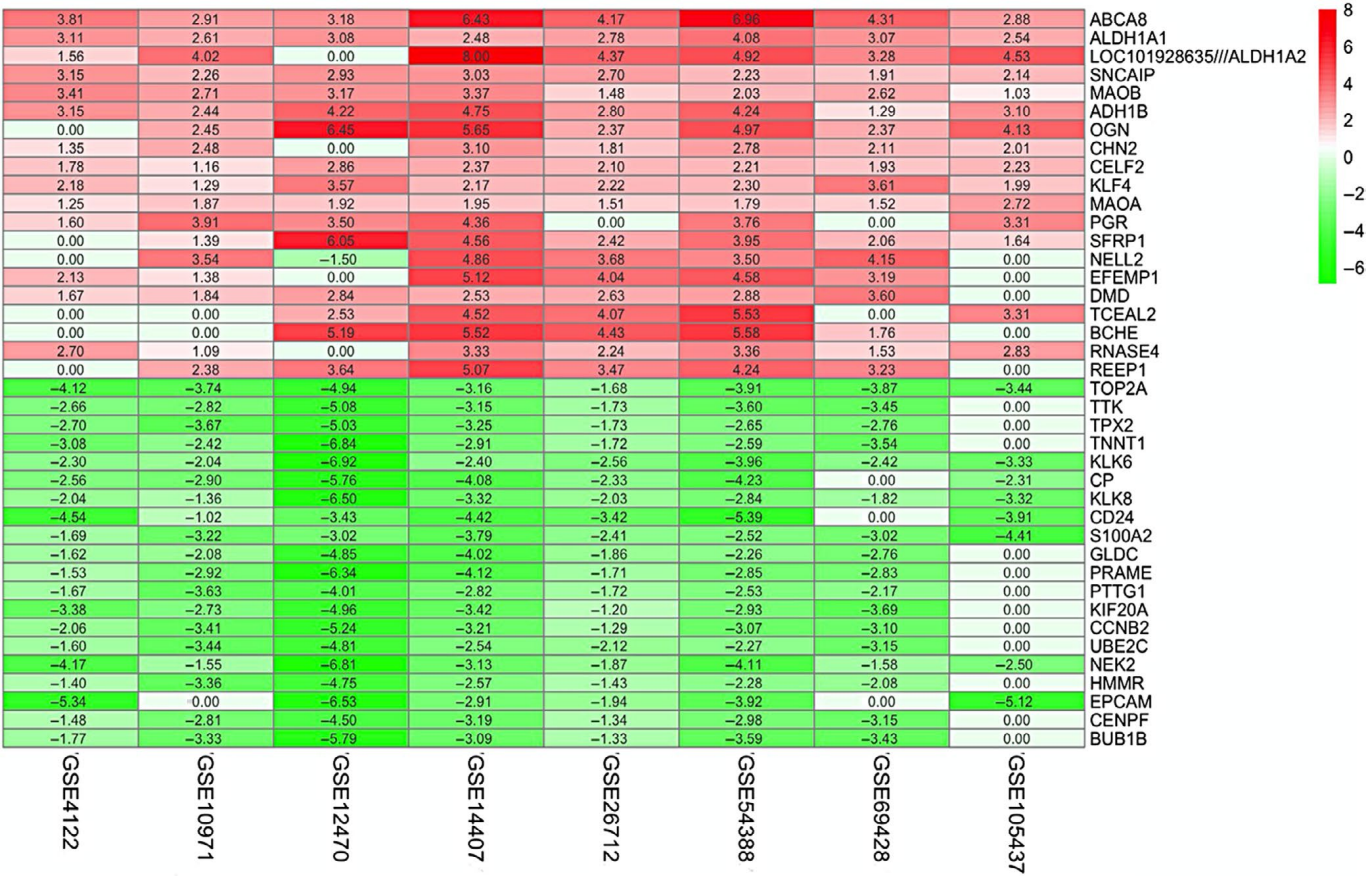
Identification of robust DEGs by RRA method. Heatmap shows the top 20 up‐regulated and down‐regulated DEGs in GEO series accessions. Each row denotes one DEG, and each column represents one data set. The colour changes from red to green indicates regulation from up to down. The numbers in the box stand for logarithmic fold change

The workflow for construction and validation of novel gene signatures for prognosis prediction in OV was shown in Figure S1. Eight eligible GEO data sets were included in the subsequent RRA analysis. The DEGs of each data set were sorted by logFC. The RRA method synthesized the ranking of genes across all data sets to determine which were selected for integrated DEGs based on the assumption that each gene in each data set was randomly arranged. In accordance with the results of RRA analysis, a total of 478 significant DEGs were identified. The top 20 up‐regulated and down‐regulated DEGs were depicted on a heatmap (Figure 1).

### 3.2 Enrichment analyses of DEGs

**FIGURE 2:**
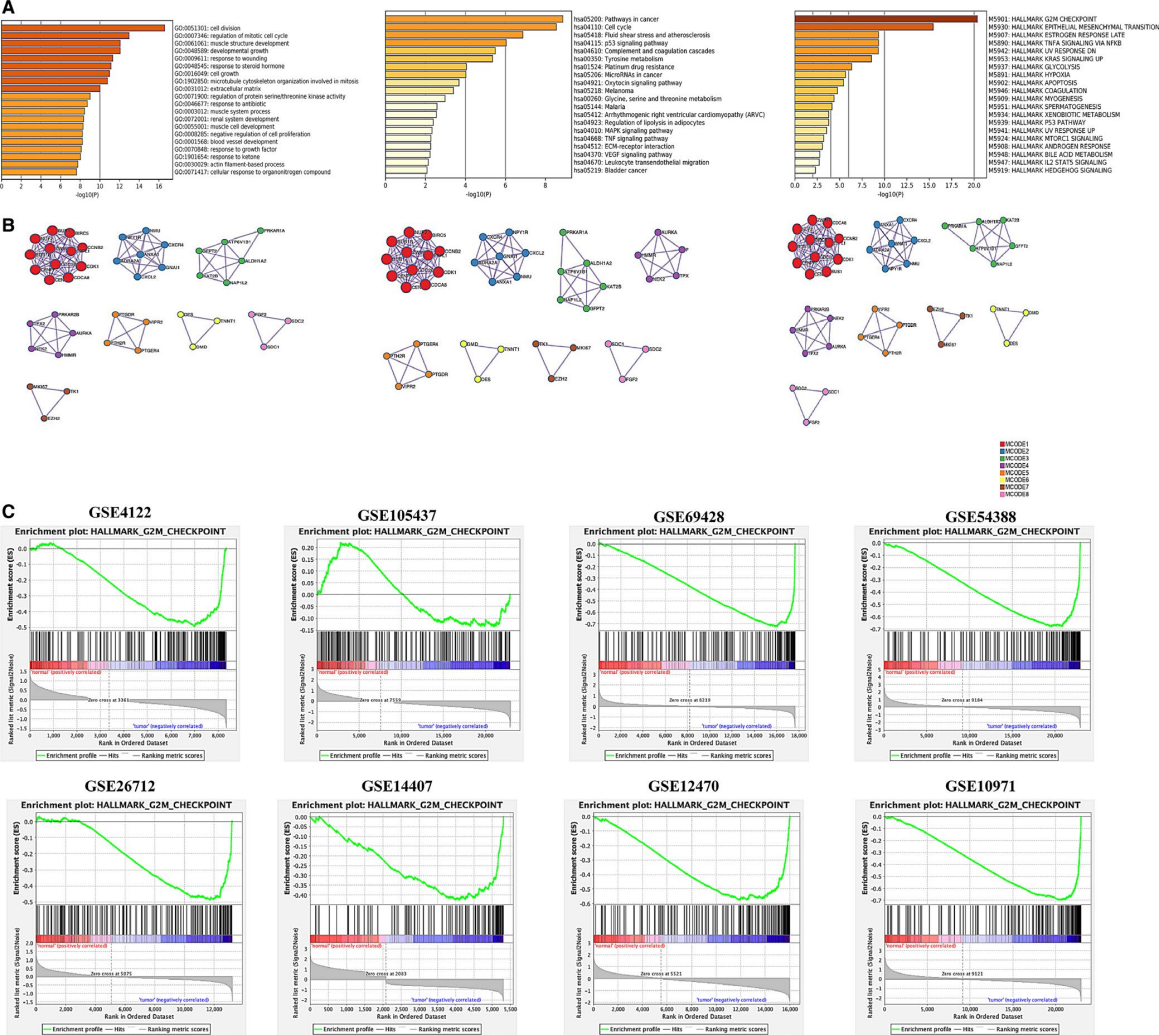
Enrichment analyses. A, Heatmaps of top 20 enriched terms across integrated DEGs, coloured by *P*‐values. B, Protein‐protein interaction network, coloured by MCODE components. Circles represent genes, and lines denote interaction between gene‐encoded proteins. C, GSEA plot of ‘HALLMARK_G2/M_CHECKPOINT’ in GSE4122, GSE105437, GSE69428, GSE54388, GSE26712, GSE14407, GSE12470 and GSE10971. The G2/M checkpoint signalling pathway is significantly suppressed in the tumour tissues compared with normal

GO annotation and KEGG pathway enrichment analysis were performed on the overall integrated DEGs. We detected that GO terms (such as cell division, regulation of mitotic cell cycle and muscle structure development) were most significantly enriched. Additionally, DEGs were significantly enriched in KEGG pathways, including pathways in cancer, cell cycle and fluid shear stress and atherosclerosis. In terms of the hallmark gene set as a reference gene set, DEGs were most significantly enriched in the following hallmark signalling pathways: ‘HALLMARK G2M CHECKPOINT’, ‘HALLMARK EPITHELIAL MESENCHYMAL TRANSITION’ and ‘HALLMARK ESTROGEN RESPONSE LATE’. The heatmaps showed top 20 significant terms of the above pathways and processes, respectively (Figure 2A). Moreover, the results of enrichment analyses were applied to each MCODE network component independently. ZWINT, ESPL1 and CDC20 were identified as hub genes in the most important modules during the process of GO and KEGG analyses, while BIRC5 replaced ZWINT as one of hub genes during the hallmark signalling pathway enrichment analysis (Figure 2B). The GSEA performance of the ‘HALLMARK G2M CHECKPOINT’ gene set revealed that it was negatively enriched in all GEO data sets when tumour tissues were compared to normal tissues (Figure 2C). In summary, G2/M checkpoint signalling pathway was most likely the vital molecular mechanism of tumorigenesis. Therefore, the genes in the hallmark gene set ‘HALLMARK G2M CHECKPOINT’ were considered as candidate biomarkers.

### 3.3 Development of the multi‐gene signature

Genes that made up the multi‐gene signature were selected from the hallmark gene set ‘HALLMARK G2M CHECKPOINT’ in the TCGA‐OV cohort by using LASSO Cox regression model. Changes in LASSO partial likelihood deviance and coefficients with log λ were shown in Figure S2. As a result, the multi‐gene signature consisted of eight genes that were highly related to prognosis in OV patients. Then, a formula was derived from coefficients and expression of genes to calculate the risk score. It was as follows: risk score = (0.0600 * CDKN1B expression) + (0.0776 * EFNA5 expression) − (0.0983 * HMGB3 expression) + (0.0072 * KATNA1 expression) − (0.0073 * MCM3 expression) + (0.0260 * PDS5B expression) + (0.0243 * SLC7A1 expression) − (0.1035 * SNRPD1 expression). Samples were subsequently divided into low‐risk and high‐risk two groups according to the median risk score.

### 3.4 Prognostic values of the multi‐gene signature

**FIGURE 3:**
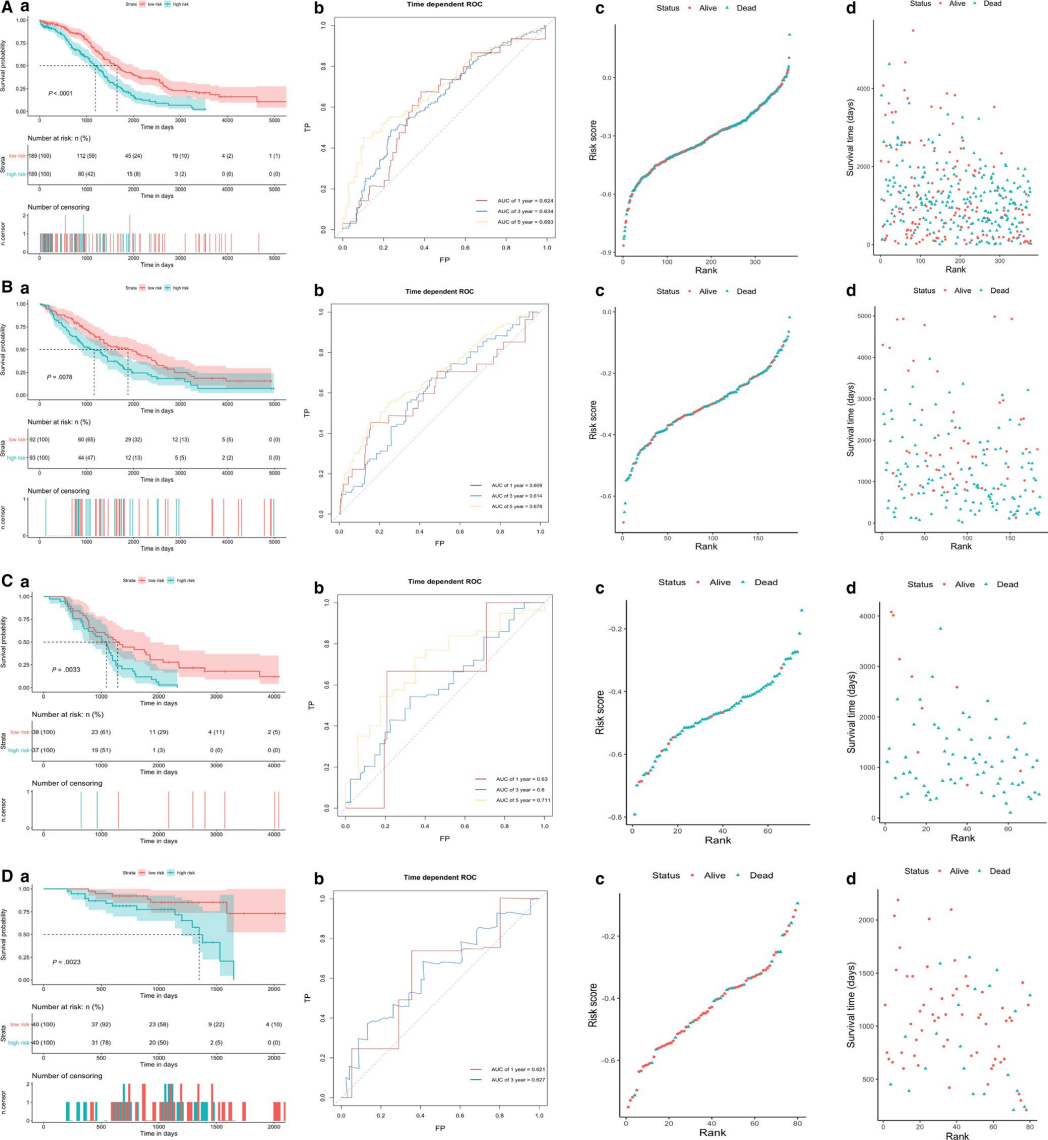
Prognostic values and predictive accuracy of the multi‐gene signature in A, TCGA‐OV data set. B, GSE26712 data set. C, GSE63885 data set. D, GSE14764 data set. a. KM survival curve (high‐risk vs low‐risk patients) for overall survival. b. Time‐dependent ROC curve for overall survival at 1, 3, 5 y. c. Risk score distribution sorted by risk rank and classified by status. d. Survival time distribution sorted by risk rank and classified by status

The risk score was ranked from low to high. In the internal data set, the distribution of risk score and survival time revealed that the patients with lower risk generally showed better survival status and longer survival time. The patients in the high‐risk group were observed poorer overall survival on the KM survival curve (*P* < .0001), which suggesting that the multi‐gene signature possessed significant prognostic value. Time‐dependent ROC analysis indicated the prognostic accuracies were 0.624 at 1 year, 0.634 at 3 year and 0.693 at 5 year, respectively (Figure 3A). In the external data set, the distribution of risk score and survival time indicated that the lower patients ranking, the better the overall survival. The result of KM survival analysis indicated overall survival was higher in low‐risk group than in high‐risk group (*P* < .0001). The prognostic accuracies at 1, 3 and 5 year in the external data sets were close to those in the internal data set (Figure 3B‐D). What's more, univariate Cox regression analysis revealed the C‐indices of our multi‐gene signature were higher than those of other biomarker‐based models in the internal and external sets, indicating better performance of our model (Table S2).

### 3.5 Validation of prognostic value in subgroups

**FIGURE 4:**
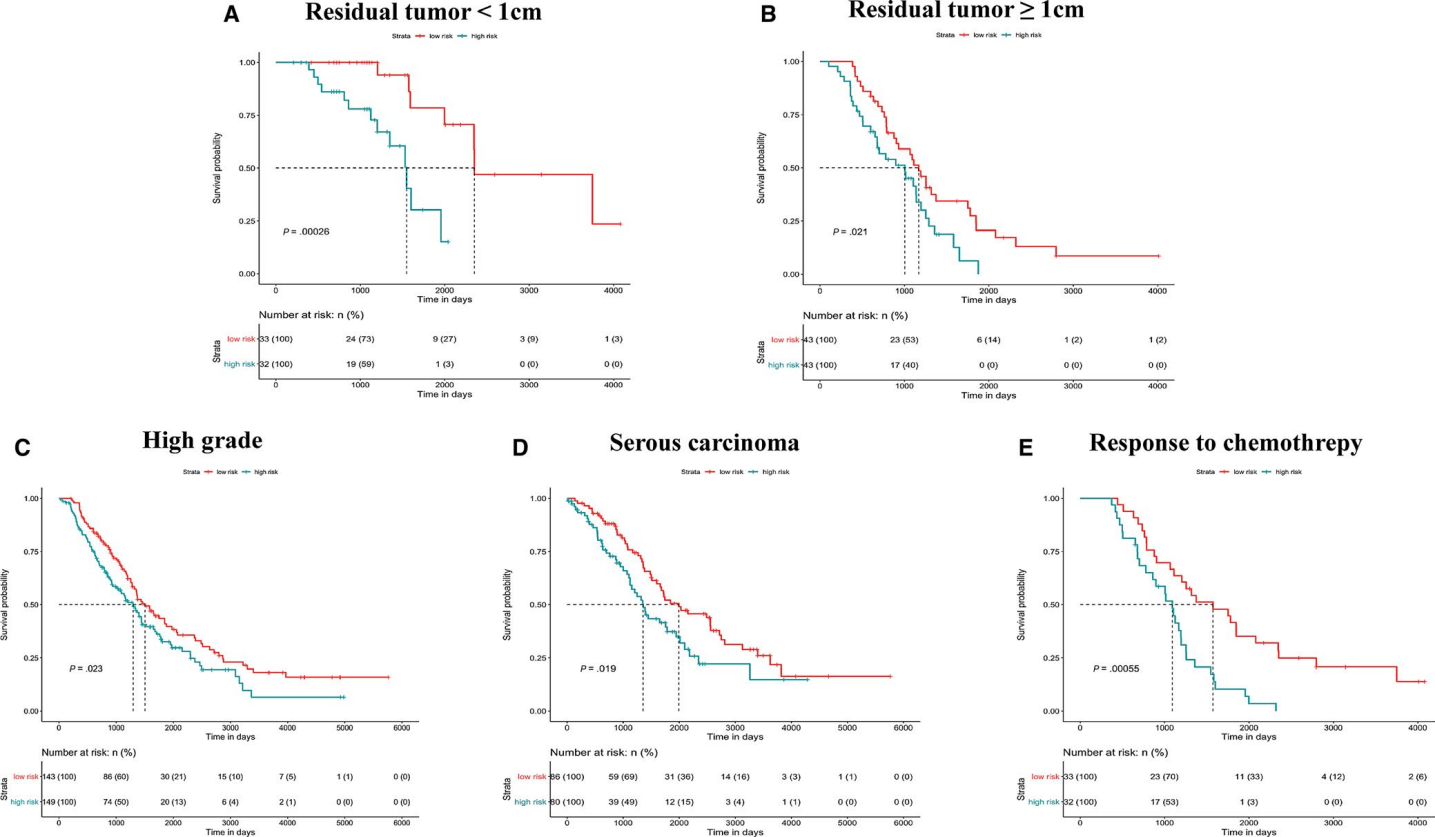
KM survival curves (high‐risk vs low‐risk patients) for overall survival in multiple subgroups. A, Residual tumour < 1 cm. B, Residual tumour ≥ 1 cm. C, High grade. D, Serous carcinoma. E, Response to chemotherapy

Subgroup analysis was performed to explore the applicability of our multi‐gene signature in predicting survival outcomes for patients with specific clinicopathological characteristics. GSE14764, GSE23554, GSE26712 and GSE63885 were integrated into a whole. Notably, all tumour samples from GSE23554 and GSE26712 were diagnosed at the advanced stage. Other detailed information of patients from these data sets was described in Table S1. According to residual tumour, patients were stratified into residual tumour <1 cm and residual tumour ≥1 cm. High grade, serous carcinoma and response to chemotherapy were the three most common subtypes of OV and the basis for categorizing other subgroups. The results of survival analysis in all subgroups showed significant differences in prognosis between low‐risk and high‐risk patients, which suggested that our prognostic model was applicable to different subtypes of OV (Figure 4).

### 3.6 Multivariate Cox regression analysis

**FIGURE 5:**
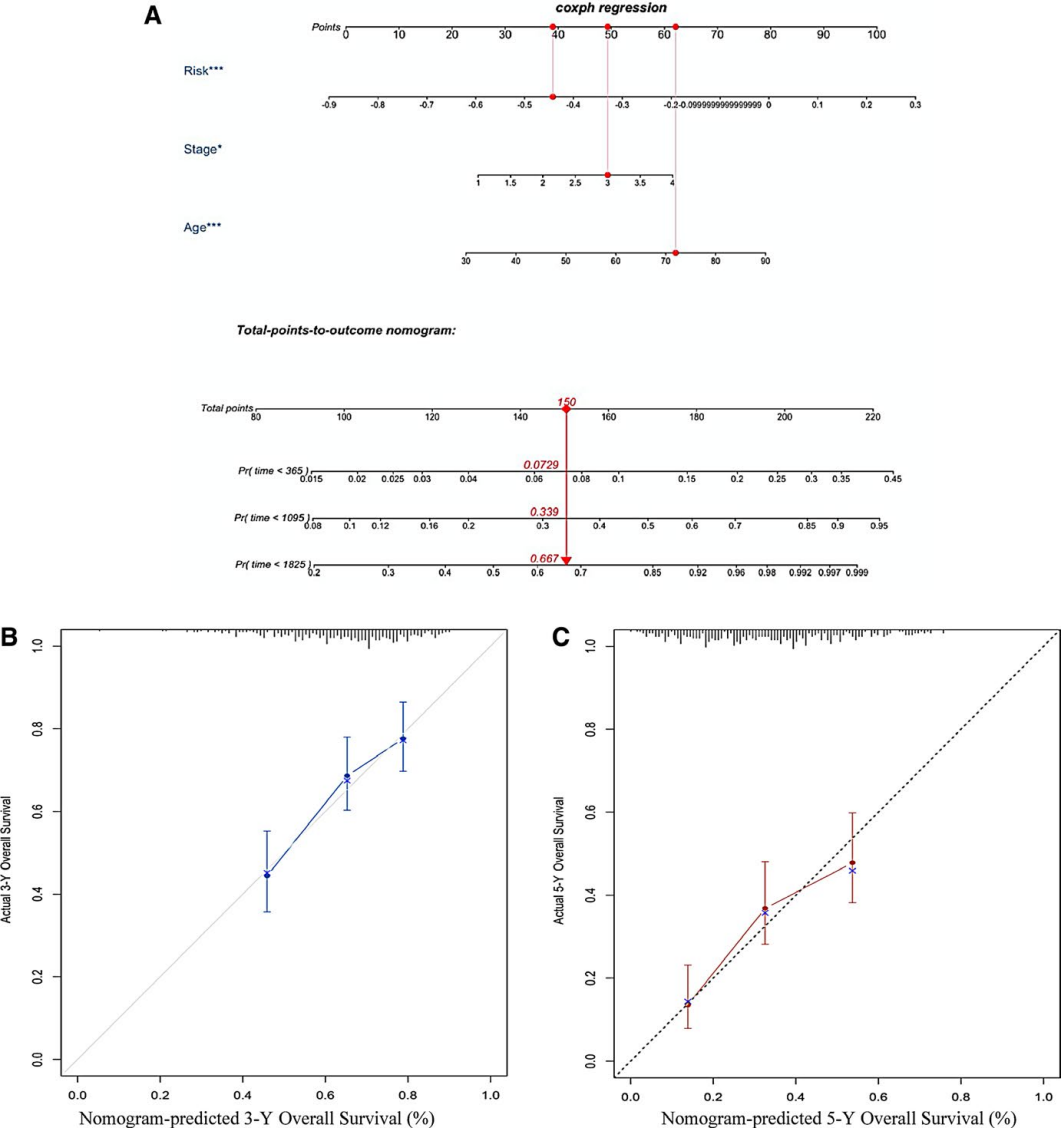
Nomogram for predicting survival probability in the TCGA data set. A, Nomogram to predict survival probability at 1, 3, 5 y. B, Calibration curve for the nomogram predicting 3‐y overall survival. C, Calibration curve for the nomogram predicting 5‐y overall survival

The result of multivariate Cox regression analysis revealed age, stage and risk score was independent factors for prognosis prediction (Figure S3). A nomogram was constructed to visualize the relationship between these independent prognostic factors and survival probability (Figure 5A). Clinicians were able to predict prognosis of patients based on their total points. Patients with higher number of total points had poorer survival outcomes. The C‐index of the nomogram was 0.695 (95% CI, 0.670‐0.727) and corrected to be 0.689 through bootstrapping validation. Furthermore, calibration curves also showed a good predictive power of the nomogram for 3‐year and 5‐year overall survival (Figure 5B,C).

### 3.7 Prognostic values of single‐gene signatures

**FIGURE 6:**
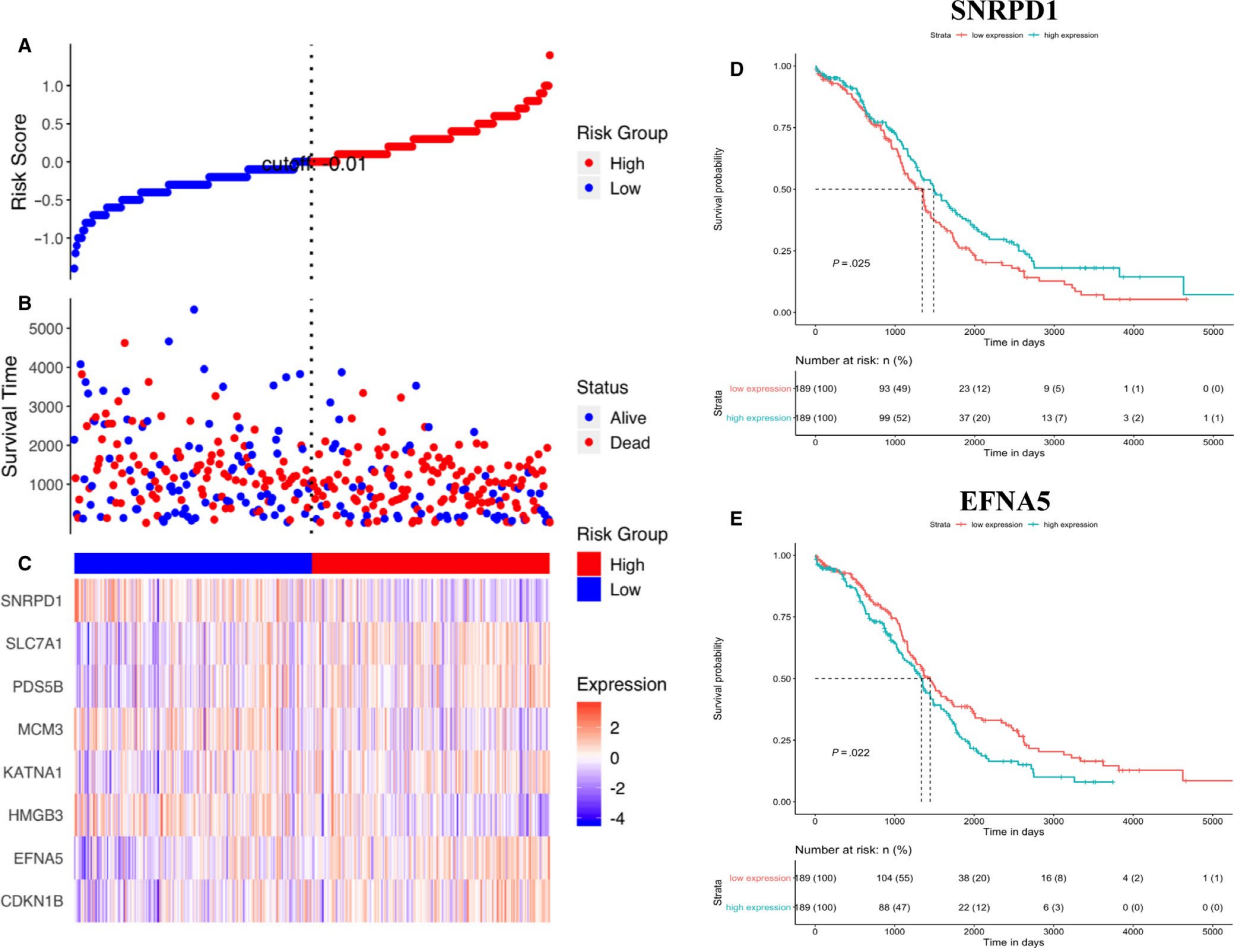
The correlation between signature gene expression and survival outcome of patients. A, Scatterplot depicts the distribution of risk scores. Each red dot indicates one high‐risk patient, and each blue dot indicates one low‐risk patient. B, Survival time distribution classified by status. C, Heatmap of gene expression. D, KM survival curve (high SNRPD1 expression vs low SNRPD1 expression patients) for overall survival. E, KM survival curve (high EFNA5 expression vs low EFNA5 expression patients) for overall survival

**FIGURE 7:**
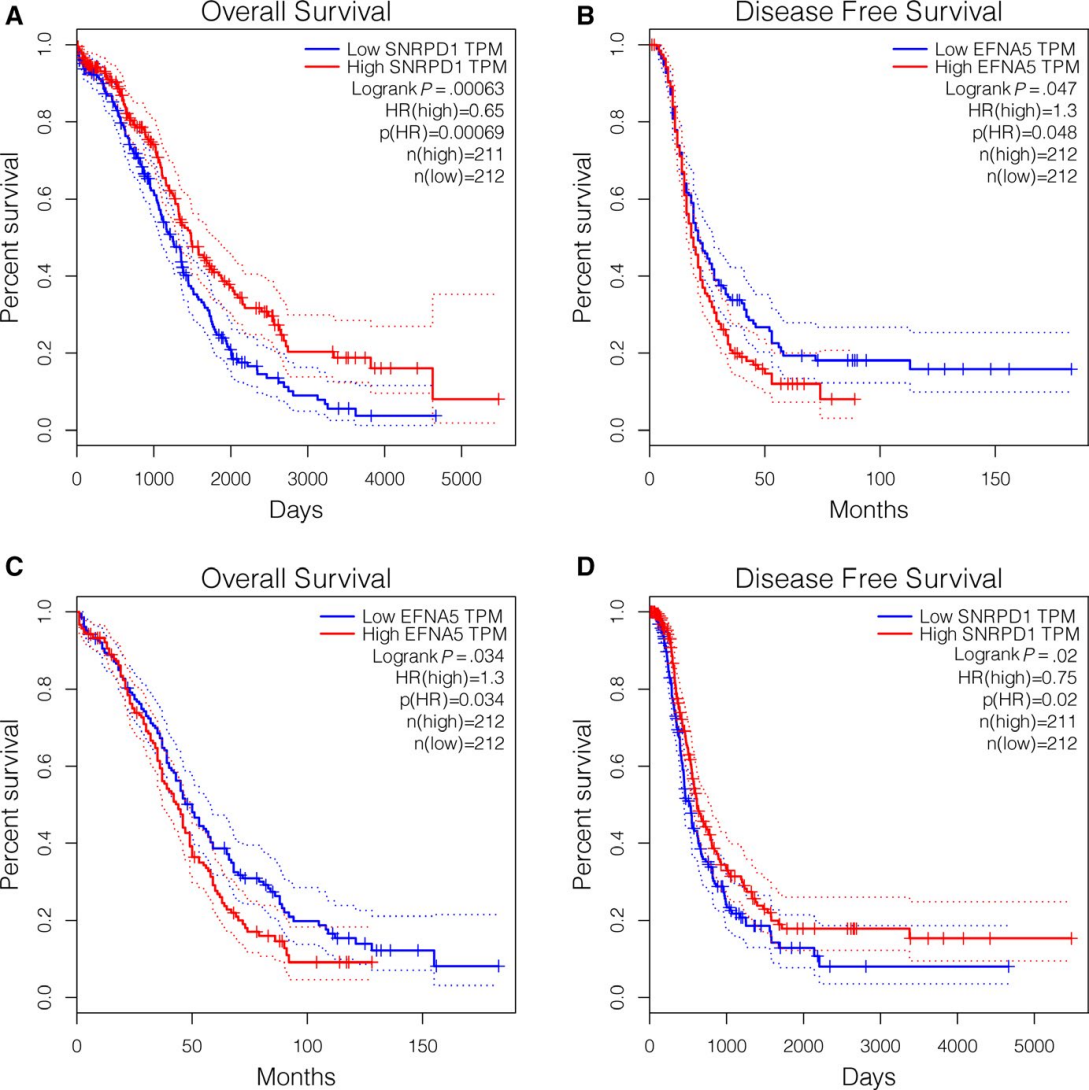
Overall survival and disease‐free survival analysis in high and low SNRPD1 and EFNA5 expression samples in the GEPIA database

It could be inferred from the high ranking of LASSO coefficients that SNRPD1 and EFNA5 played important roles in the multi‐gene signature. The most robust correlations between their expression and risk stratification among all eight members of the multi‐gene signature were observed (Figure 6A‐C). In addition, the patients were divided into two groups by the median expression. Survival analysis showed that patients with lower expression of SNRPD1 had significantly poorer overall survival and higher EFNA5 expression indicated poorer outcome (Figure 6D,E). Significant differences in prognosis between high and low expression patients revealed prognostic value of the gene expression. Therefore, two single‐gene signatures were built based on SNRPD1 expression and EFNA5 expression as simplified supplements to the multi‐gene signature in clinical applications. We conducted differential expression and KM survival analyses to further assess prognostic value of single‐gene signatures. As a result, the expression of SNRPD1 in tumour tissues was higher than that in normal tissues. Significant increase of SNRPD1 expression in different subtypes of OV was also observed based on the Oncomine database (Figure S4). In the GEPIA set, SNRPD1 expression showed significant association with overall survival and disease‐free survival (Figure 7A,B), which confirmed its prognostic value. Similar results were obtained from performance of the same analyses on EFNA5 expression, and the prognostic value was validated (Figure 7C,D and Figure S5). Notably, it was higher EFNA5 expression and lower SNRPD1 that predicted poorer prognosis. At last, univariate and multivariable Cox regression model identified both single‐gene signatures as independent prognostic factors for patients with OV (Table 2).

**TABLE 2:** Univariate and multivariate Cox regression model in predicting overall survival of ovarian cancer

Characteristics	Univariate model		Multivariate model	
	HR (95% CI)	*P*‐value	HR (95% CI)	*P*‐value
Age	1.021 (1.009‐1.034)	<.001	1.022 (1.010‐1.035)	<.001
FIGO_stage				
I/II	Reference	.060	Reference	.064
III/IV	2.183 (0.969‐4.919)		2.161 (0.956‐4.884)	
SNRPD1 expression				
Low	Reference	.026	Reference	.013
High	0.745 (0.575‐0.965)		0.715 (0.548‐0.932)	
EFNA5 expression				
Low	Reference	.022	Reference	.002
High	1.355 (1.044‐1.759)		1.532 (1.170‐2.006)	

Abbreviations: CI, confidence interval; FIGO, International Federation of Gynecology and Obstetrics; HR, hazard ratio.

### 3.8 Correlations between signature genes expression and clinical traits

Clinical information of OV samples, such as stage, age, living status and survival time, was incorporated with expression of DEGs selected from RRA analysis. A total of eight clinical traits‐related modules were generated with a soft‐thresholding power of 9 and a cut height of 0.25. The majority of genes in ‘Hallmark G2/M checkpoint’ gene set were divided into black modules that was significantly associated with tumour stage. Among them, five genes with high levels of MM and GS were the members of multi‐gene signatures, including SNRPD1, SLC7A1, PDS5B, MCM3 and HMGB3. Besides, EFNA5, CDKN1B and KATNA1 were identified as members of turquoise module which was significantly related to living status (Figure S6).

### 3.9 Drug‐gene interaction

CDKN1B and SLC7A1 were identified as promising targets for potential drug reactions based on the results of drug‐gene interaction exploration using DGIdb (Table 3). A total of sixteen candidate drugs were searched out, eleven of which had been approved by the Food and Drug Administration (FDA). The majority of potential drugs were likely to interact with the CDKN1B, as shown on the network of signature genes performance. CDKN1B might have downstream effects on minichromosome maintenance complex component family, phosphate, bortezomib and rapamycin (Figure S7).

**TABLE 3:** Potential drugs interacted with the signature genes

Gene	Drug	Sources	PMIDs	FDA approval
CDKN1B	METHOTREXATE	NCI	14512390	No
CDKN1B	EPOETIN ALFA	NCI	15122318	Yes
CDKN1B	RALTITREXED	NCI	10047461	Yes
CDKN1B	CHEMBL35482	NCI	11031257	No
CDKN1B	EPOETIN BETA	NCI	11023508	Yes
CDKN1B	TRETINOIN	NCI	10837916	Yes
CDKN1B	PROXYPHYLLINE	NCI	12097373	Yes
CDKN1B	DOXORUBICIN	NCI	12576455	Yes
CDKN1B	CELECOXIB	PharmGKB		No
CDKN1B	STREPTOZOTOCIN	NCI	11978652	Yes
CDKN1B	VINCRISTINE	NCI	12576455	Yes
CDKN1B	LAPATINIB	CIViC	25587029	Yes
CDKN1B	PROGESTERONE	NCI	11590147	Yes
SLC7A1	LYSINE	DrugBank	17042743; 9614060; 14523001; 17139284; 17016423	No
SLC7A1	ORNITHINE	DrugBank	15491978; 16703566; 17065601	Yes
SLC7A1	ARGININE	DrugBank	17329401; 17427197; 17363779; 17325243; 17065601	No

Abbreviation: FDA, Food and Drug Administration.

## 4 DISCUSSION

A multi‐gene signature was constructed through multi‐step bioinformatic analysis. First, we identified DEGs between tumour and normal tissues in several GEO databases and integrated the results using the RRA method. Next, G2/M checkpoint ranked the top significant hallmark gene set in which DEGs were enriched according to enrichment analyses. Moreover, a correlation between G2/M checkpoint signalling pathway and tumour progression was observed based on the performance of GSEA. Consequently, the genes related to G2/M checkpoint were selected as candidate biomarkers. Finally, the LASSO Cox regression model was applied to build a multi‐gene signature and its prognostic value was further validated in different databases and common subtypes of OV.

OV is a common gynaecological tumour with a heterogeneous category. The specific cell of origin divides OV into epithelial and non‐epithelial cancers and the former type accounts for approximately ninety per cent of OV.^28^ Epithelial OVs (EOC) are further categorized as four main histologic subtypes: serous, endometrioid, mucinous and clear cell, with minority classified as rare and undifferentiable subtypes.^14^ Serous and endometrioid EOCs share an additional stratification of tumour grade according to the apparent degree of cytological aberration.^29^ Modes of carcinogenesis, molecular‐genetic features and sites of origin distinguish between high‐grade and low‐grade serous carcinomas.^30^, ^31^, ^32^ Recent classification on the basis of the dualistic model segregates EOCs into type I and type II from the clinicopathological and molecular prospective.^16^ Type II tumours are considered high grade, diagnosis at advanced stage and low survival, wherefore result in the major fraction of OV deaths. One of the important factors in elevating the mortality of OV patients is that effective screening tests remain blank to date. A recent large randomized trial combining transvaginal ultrasound with changes in CA125 has observed a reduction in mortality after long‐term follow‐up but screening strategies based on secondary analysis remain controversial.^33^, ^34^

In addition, current treatment of OV is limited to radical surgery and chemotherapy, which prolongs the interval between recurrences but does not benefit overall survival.^35^ A variety of approaches to management of OV should be developed to target different subtypes with varying survival rates. It is of vital importance to establish effective prognostic predictors to guide treatment choices for patients. Although a few standard phenotypes, such as tumour stage and grade, have been applied to decide whether a patient should be recommended to undergo adjuvant chemotherapy after cytoreductive surgery, it is not enough to distinguish patients at increased risks of tumour progression.^28^ Therefore, more emphasis should be placed on molecular mechanisms to reveal the main factors associated with clinical outcomes.

Fortunately, rapid advances in genome sequencing and integrated bioinformatics have provided opportunities to discover molecular biomarkers with prognostic value for OV. The predictive value of multi‐gene signatures has been highlighted in many other cancer types.^36^, ^37^, ^38^, ^39^ Nevertheless, recent researches on prognosis prediction are limited to the most common types of OV, namely high‐grade serous OV.^17^ Therefore, there is an urgent need for a more widely used model. In our study, the included data sets contained samples of all categories so that robust integrated DEGs covered the differences between various types of tumours and normal tissues. Consequently, the multi‐gene signature constructed on the basis of these DEGs has more extensive applications in clinical practice. By incorporating the multi‐gene signature and clinical prognostic variables, a visual nomogram was established for quantitatively predicting 1‐, 3‐ and 5‐year overall survival of OV patients. Additionally, the prognostic value of the multi‐gene signature was validated in all subgroups, indicating the independence from clinicopathological factors. The larger C‐indices of our multi‐gene signature demonstrated better performance for survival prediction than other biomarker‐based predictors.

However, applying the multi‐gene signature for prognosis prediction costs more medical expenses. Since it has been reported that many single‐molecule biomarkers are also related to clinical outcomes, we simplified the model into two single‐gene markers at the cost of prediction accuracy to improve practicality. Both SNRPD1 and EFNA5 are members of the ‘HALLMARK G2M CHECKPOINT’ gene set, so they participate in cell proliferation and tumour progression via cell cycle arrest at the G2/M‐phase. Furthermore, SNRPD1 plays an important role in manipulating the regulation of pluripotency‐specific spliceosome assembly and the acquisition and maintenance of pluripotency.^40^ SNRPD1 has also been reported to be involved in osteogenic differentiation of mesenchymal stem cell.^41^ Regarding the function of EFNA5, recent studies have shown its significant expression alterations in prostate cancer, gastric cancer and colorectal cancer compared to conventional normal tissues.^42^, ^43^, ^44^ Notably, EFNA5 is likely to be one of novel candidate genes that contribute to human Mendelian disorders.^45^ The above findings also indicate that the change in EFNA5 expression seems to be applicable to a variety of genetic diseases and has low specificity for OV.

Previous studies have applied pathological stage as an indicator to guide treatment choice but little direct evidence suggested it could be regarded as an accurate factor for predicting the prognosis of OV patients.^16^, ^28^ Nevertheless, tumour stage was identified as an independent predictor in our nomogram. Interestingly, the prognostic prediction ability of tumour stage decreased in the multivariate Cox regression combined with two single‐gene signatures, which possibly resulted from the correlation between SNRPD1 expression and tumour stage, as revealed by the process of WGCNA.

In conclusion, our study constructed novel gene signatures for prognosis prediction in OV based on the G2/M checkpoint signalling pathway enrichment. Prognostic value of the multi‐gene signature was validated in the internal, external and entire sets. Independence from other clinical factors was determined through subgroup analysis. By incorporating this signature with age and pathological tumour stage, a visual predictive nomogram was established, which was convenient for predicting survival outcomes of OV patients. Two single‐gene signatures were also built as simplified independent prognostic factors to satisfy diversified clinical requirements. However, these models require further verification in different clinical centres in the future.

## CONFLICT OF INTEREST

The authors declare no conflict of interest.

## AUTHOR CONTRIBUTIONS

**Mingyang Bao:** Conceptualization (equal); data curation (lead); formal analysis (lead); investigation (equal); methodology (lead); project administration (equal); resources (equal); software (lead); supervision (supporting); validation (equal); visualization (lead); writing – original draft (lead); writing – review & editing (equal). **Lihua Zhang:** Conceptualization (equal); data curation (supporting); formal analysis (supporting); investigation (equal); methodology (supporting); project administration (supporting); resources (supporting); software (supporting); supervision (supporting); validation (supporting); visualization (supporting); writing – original draft (supporting); writing – review & editing (supporting). **Yueqing Hu:** Conceptualization (equal); funding acquisition (lead); investigation (equal); methodology (supporting); project administration (equal); supervision (equal); validation (equal); writing – review & editing (equal).

## Supporting information

Fig S1

Fig S2

Fig S3

Fig S4

Fig S5

Fig S6

Fig S7

Table S1

Table S2

## Data Availability

The data sets used and/or analysed during the current study are available from the GEO database (http://www.ncbi.nlm.nih.gov/geo/) and TCGA database (https://cancergenome.nih.gov/).
