# Supplementary figures and images for: Novel gene signatures for prognosis prediction in ovarian cancer
Source: J Cell Mol Med. 2020 Jul 14;24(17):9972–84. doi: 10.1111/jcmm.15601 (PMC7520318; doi:10.1111/jcmm.15601)

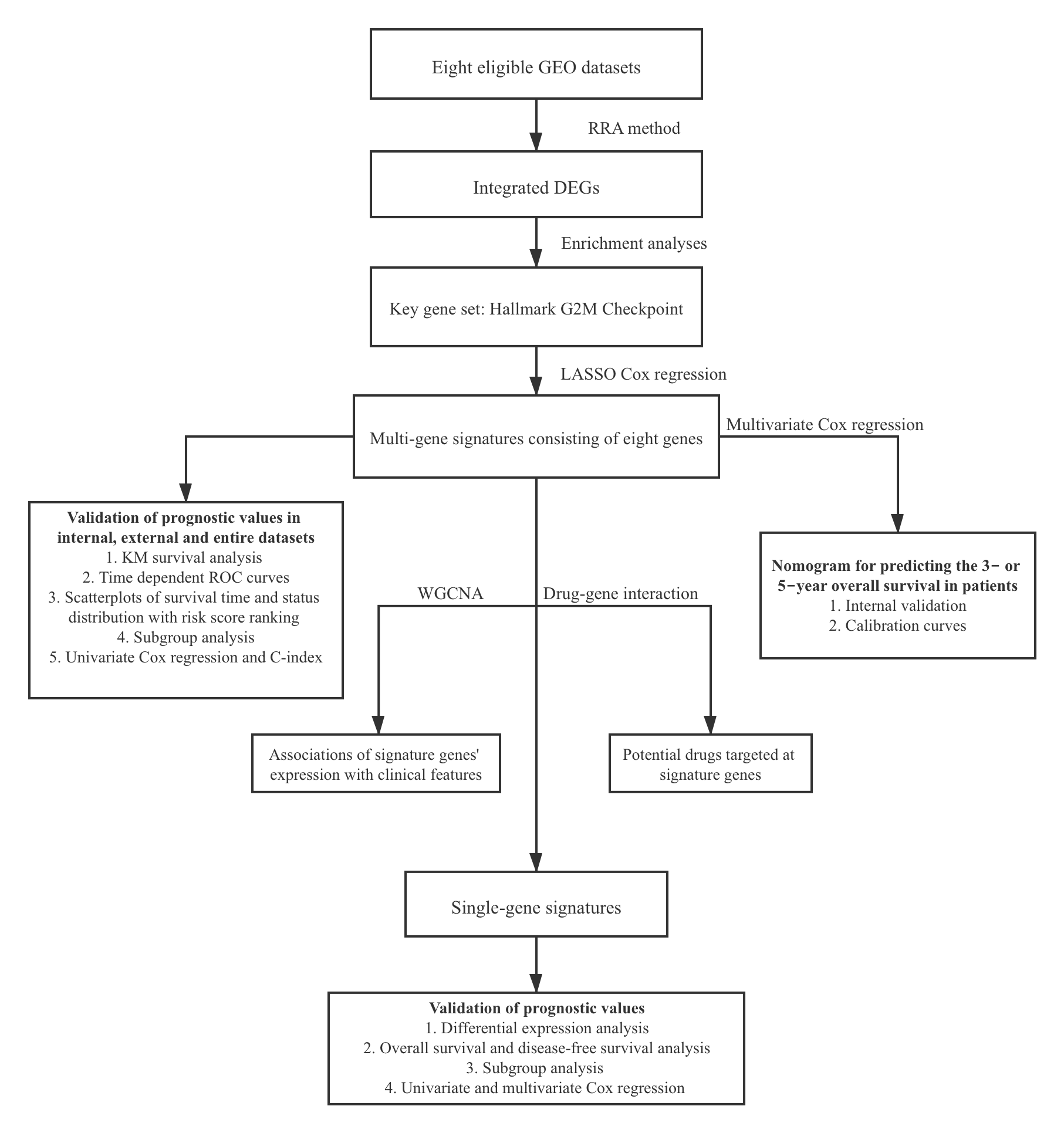

Supplement: Supplementary file 1 — Fig S1 [file JCMM-24-9972-s001.tif]

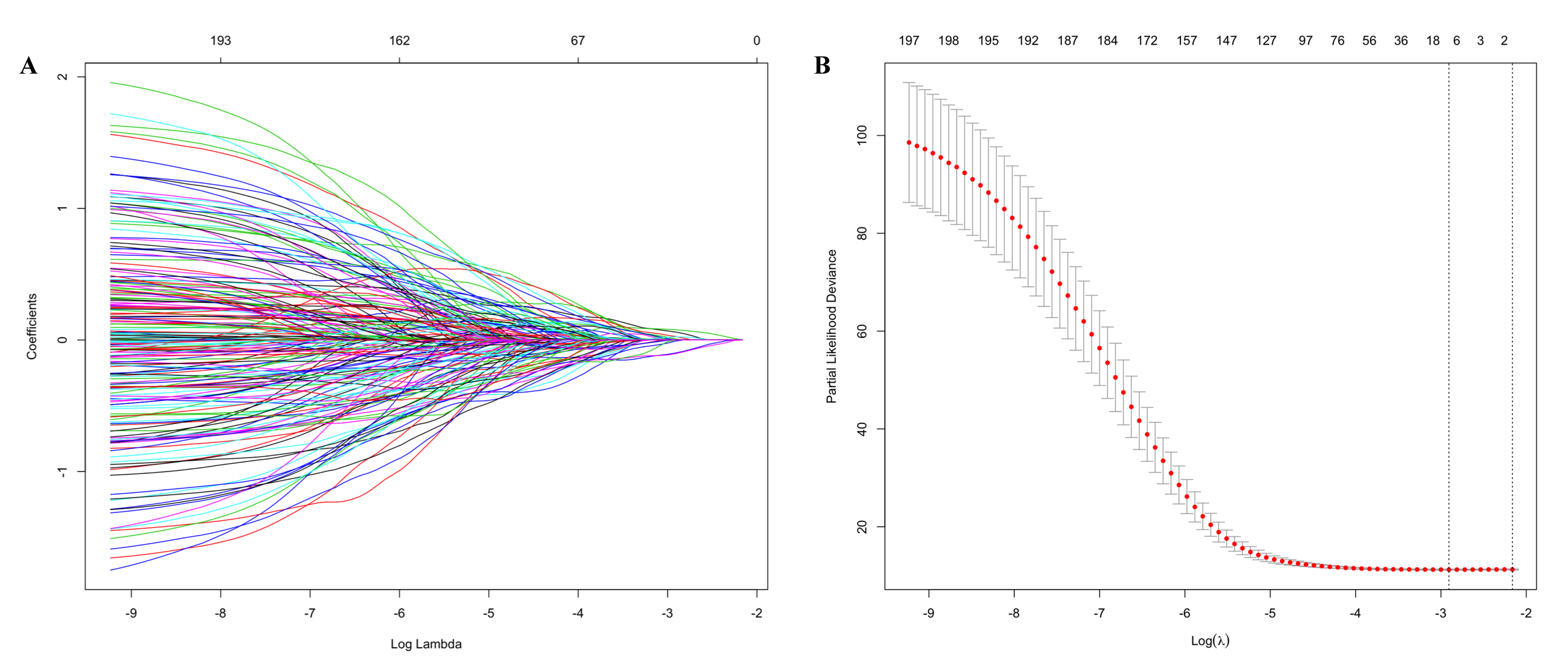

Supplement: Supplementary file 2 — Fig S2 [file JCMM-24-9972-s002.tif]

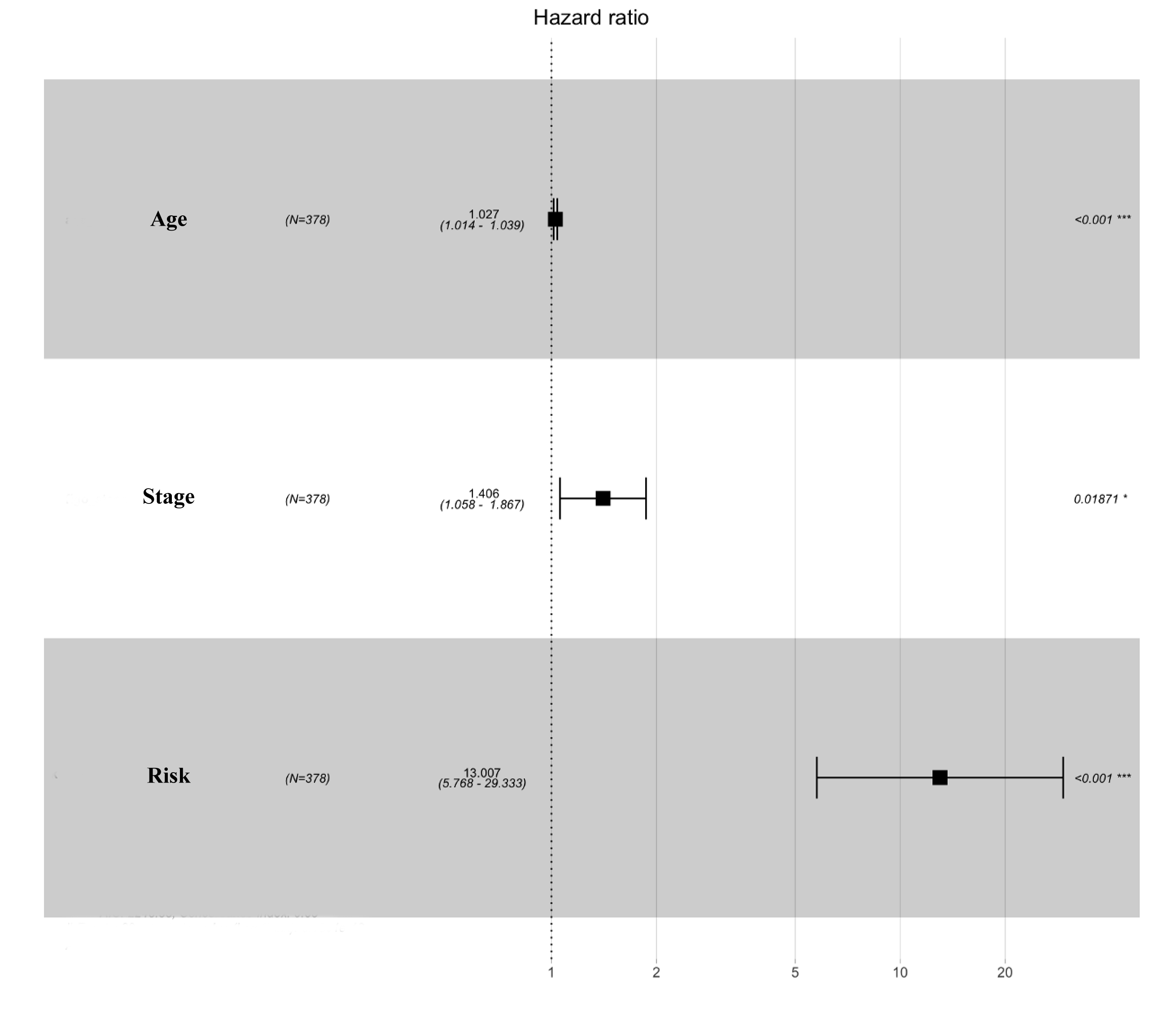

Supplement: Supplementary file 3 — Fig S3 [file JCMM-24-9972-s003.tif]

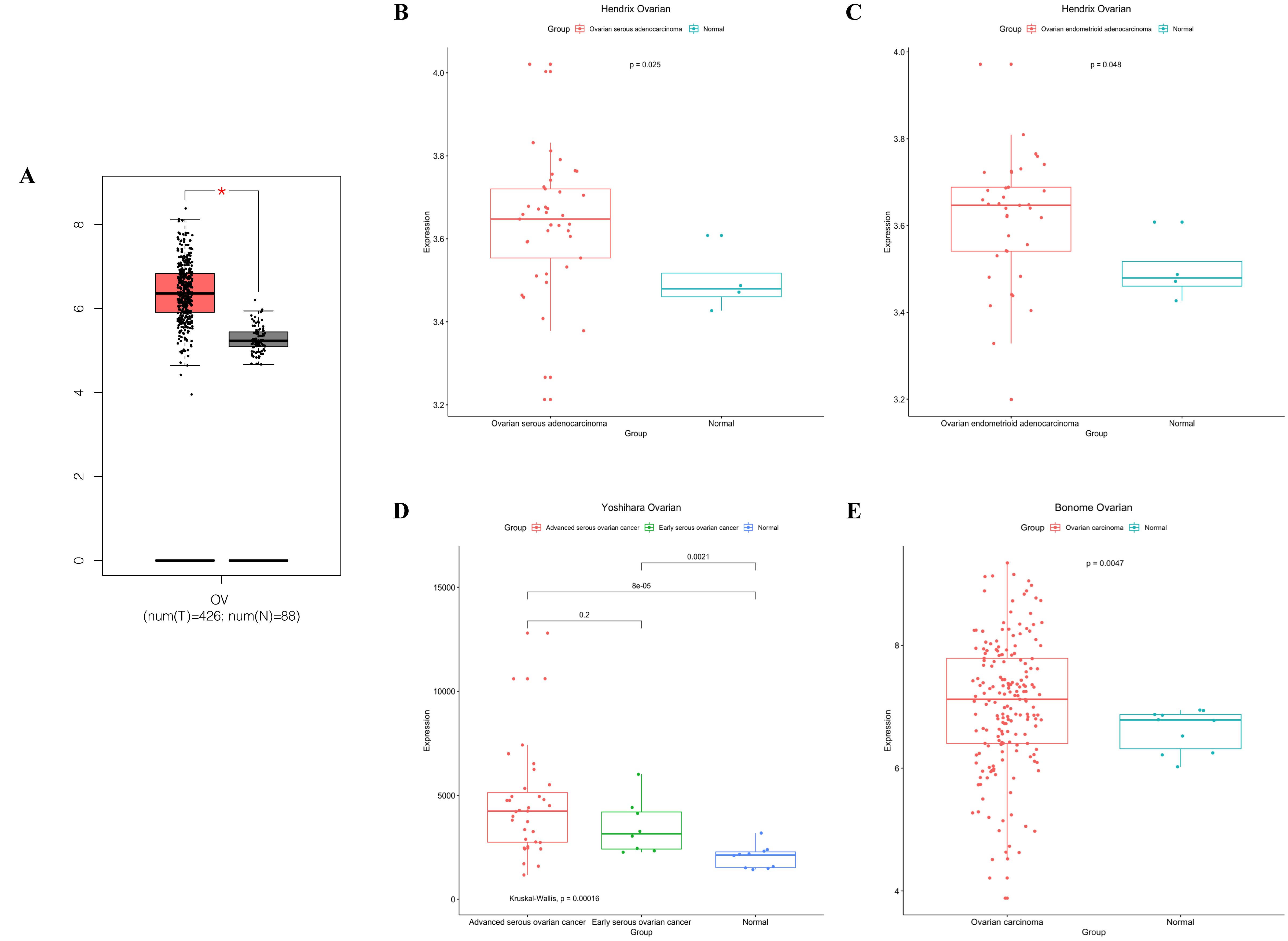

Supplement: Supplementary file 4 — Fig S4 [file JCMM-24-9972-s004.tif]

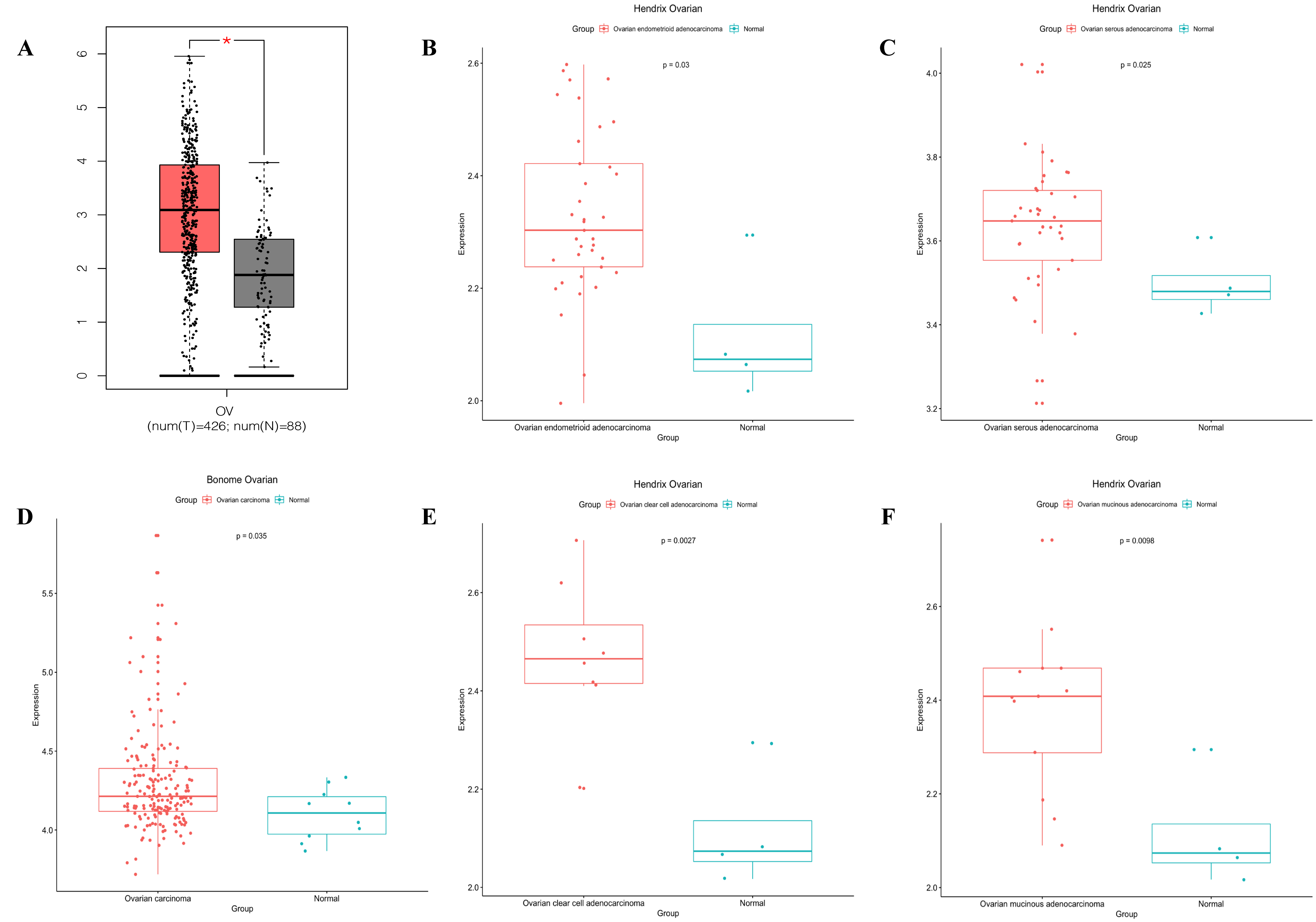

Supplement: Supplementary file 5 — Fig S5 [file JCMM-24-9972-s005.tif]

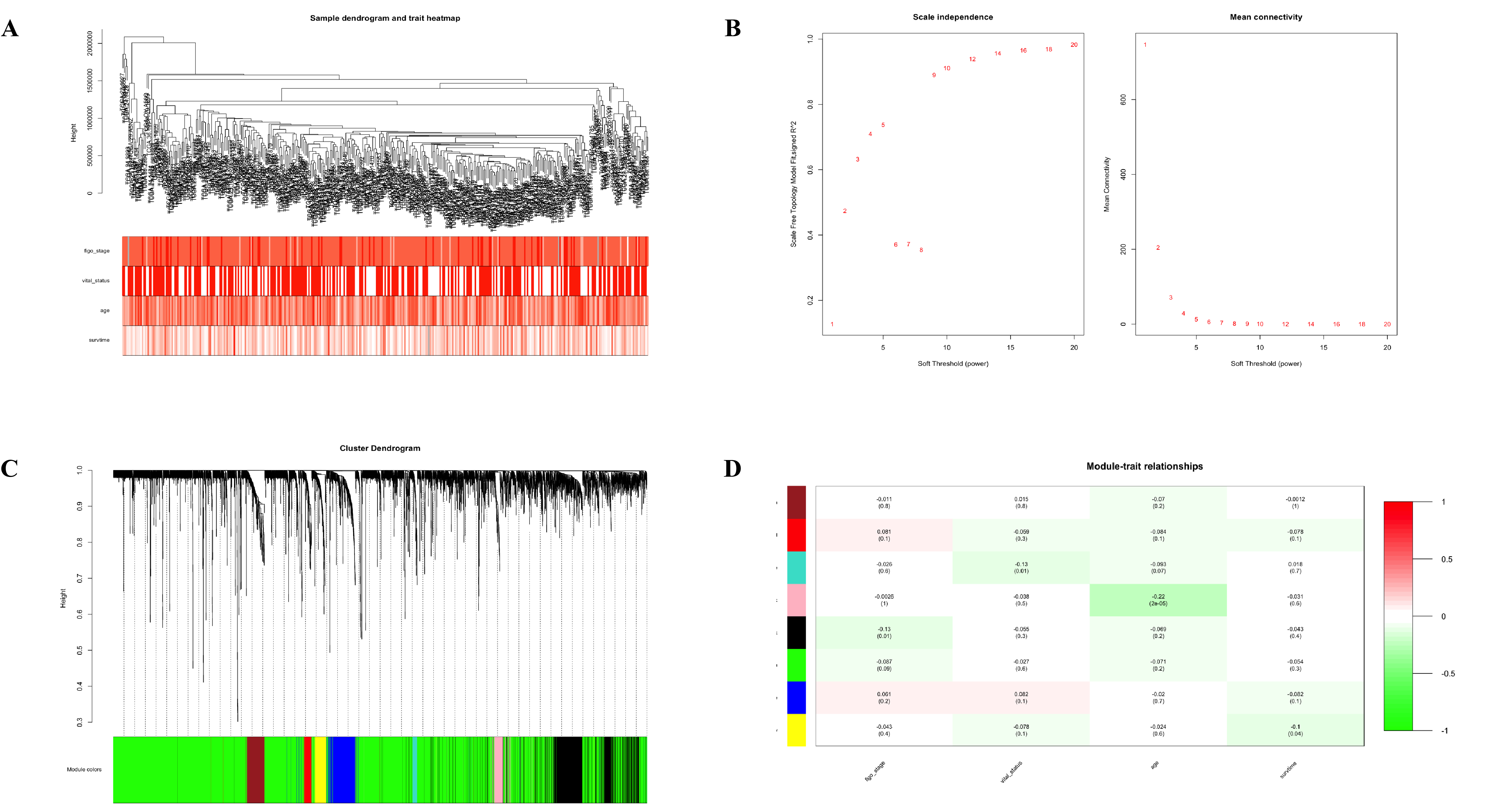

Supplement: Supplementary file 6 — Fig S6 [file JCMM-24-9972-s006.tif]

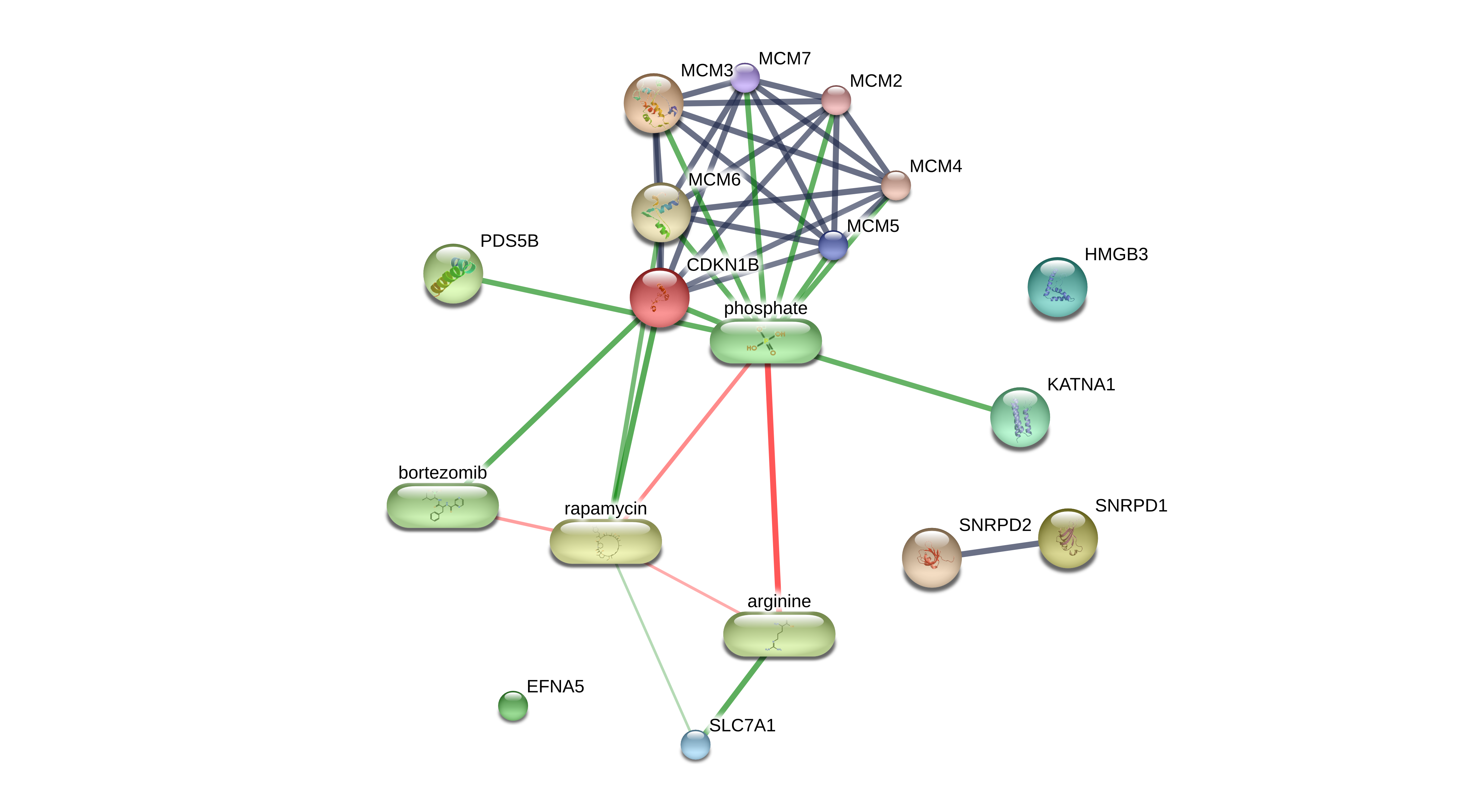

Supplement: Supplementary file 7 — Fig S7 [file JCMM-24-9972-s007.tif]
