# Supplementary material for: Novel gene signatures for prognosis prediction in ovarian cancer
Source: J Cell Mol Med. 2020 Jul 14;24(17):9972–84. doi: 10.1111/jcmm.15601 (PMC7520318; doi:10.1111/jcmm.15601)
Supplement: Supplementary file 8 — Table S1 [file JCMM-24-9972-s008.docx]

Table S1 Clinical characteristics of patients from the datasets applied in subgroup analysis

| **Characteristic** | **TCGA-OV cohort** | | **P-value** | **GSE 23554** | | **P-value** | **GSE 14764** | | **P-value** | **GSE 63885** | | **P-value** |
| --- | --- | --- | --- | --- | --- | --- | --- | --- | --- | --- | --- | --- |
|  | **Alive (n=146)** | **Dead** |  | **Alive (n=14)** | **Dead** |  | **Alive (n=59)** | **Dead** |  | **Alive (n=9)** | **Dead** |  |
|  |  | **(n=232)** |  |  | **(n=14)** |  |  | **(n=21)** |  |  | **(n=66)** |  |
| **Age** |  |  | 0.090 |  |  | - |  |  | - |  |  | - |
| < 60 | 85(58.2%) | 113(48.7%) |  | - | |  | - | |  | - | |  |
| ≥ 60 | 61(41.8%) | 119(51.3%) |  |  |  |  |  |  |  |  |  |  |
| **Grade** |  |  | - |  |  | 1.000 |  |  | 0.860 |  |  | 0.600 |
| High Grade | - | |  | 9 (64.3%) | 9 (64.3%) |  | 39(66.1%) | 15(71.4%) |  | 6(66.7%) | 42(63.6%) |  |
| Low Grade |  |  |  | 5 (35.7%) | 5(35.7%) |  | 20(33.9%) | 6(28.6%) |  | 2(22.2%) | 7(10.6%) |  |
| **Histology** |  |  | 1.000 |  |  | 1.000 |  |  | 0.502 |  |  | 0.482 |
| Serous | 146(100%) | 232(100%) |  | 14(100%) | 14(100%) |  | 49(83.1%) | 19(90.5%) |  | 8(88.9%) | 62(93.9%) |  |
| Not serous | 0(0.0%) | 0(0.0%) |  | 0 (0.0%) | 0 (0.0%) |  | 10(26.9%) | 2(9.5%) |  | 1(11.1%) | 4(6.1%) |  |
| **FIGO_stage** |  |  | < 0.001 |  |  | - |  |  | 0.433 |  |  | 0.227 |
| I/II | 20(13.7%) | 7(3.0%) |  | - | |  | 8(13.6%) | 1(4.8%) |  | 1(11.1%) | 1(1.5%) |  |
| III/IV | 126(86.3%) | 225(97.0%) |  |  |  |  | 51(86.4%) | 20(95.2%) |  | 8(88.9%) | 65(98.5%) |  |
