# Supplementary material for: Novel gene signatures for prognosis prediction in ovarian cancer
Source: J Cell Mol Med. 2020 Jul 14;24(17):9972–84. doi: 10.1111/jcmm.15601 (PMC7520318; doi:10.1111/jcmm.15601)
Supplement: Supplementary file 9 — Table S2 [file JCMM-24-9972-s009.docx]

Table S2 C-indices for our multi-gene signature and other biomarkers-based models

| Biomarkers-based model | Internal set | | External set | |
| --- | --- | --- | --- | --- |
|  | C-index (95% CI) | | C-index (95% CI) | |
| BIRC5 | 0.536 | (0.496~0.577) | 0.513 | (0.454~0.572) |
| BUB1B | 0.512 | (0.470~0.553) | 0.521 | (0.462~0.579) |
| CCNB2 | 0.489 | (0.450~0.529) | 0.511 | (0.454~0.569) |
| CDC20 | 0.495 | (0.455~0.535) | 0.535 | (0.476~0.594) |
| FOXM1 | 0.497 | (0.456~0.537) | 0.503 | (0.445~0.561) |
| KIF11 | 0.514 | (0.473~0.556) | 0.500 | (0.442~0.557) |
| KIF4A | 0.503 | (0.463~0.543) | 0.518 | (0.460~0.577) |
| TYMS | 0.513 | (0.473~0.554) | 0.556 | (0.499~0.613) |
| Tumor microenvironment-related multi-gene signature | 0.610 | (0.571~0.649) | 0.581 | (0.524~0.638) |
| **G2M checkpoint-related multi-gene signature** | 0.647 | (0.608~0.686) | 0.602 | (0.546~0.658) |

CI, confidence interval
